# Normal limits for left ventricular ejection fraction and volumes determined by gated single photon emission computed tomography – a comparison between two quantification methods

**DOI:** 10.1111/j.1475-097X.2008.00791.x

**Published:** 2008-05

**Authors:** Milan Lomsky, Lena Johansson, Peter Gjertsson, Jonas Björk, Lars Edenbrandt

**Affiliations:** 1 Department of Clinical Physiology, Sahlgrenska University HospitalGöteborg, Sweden; 2 Competence Centre for Clinical Research, Lund University HospitalLund, Sweden; 3 Department of Clinical Sciences, Lund University Research Program in Medical Informatics, Malmö University HospitalMalmö, Sweden

**Keywords:** *computer-assisted*, *gender*, *heart function tests*, *radionuclide imaging*, *reference values*

## Abstract

To compare gender-related normal limits for left ventricular (LV) ejection fraction (EF), end-diastolic and end-systolic volumes (EDV and ESV), obtained using two myocardial perfusion-gated single photon emission computed tomography (SPECT) quantification methods. A total of 185 patients were retrospectively selected from a consecutive series of patients examined for coronary artery disease (CAD) or for management of known CAD. Patients were included in the study group if they had normal or probably normal results with stress and rest perfusion imaging and if the combined interpretation of perfusion studies and gated rest studies showed no signs or suspicion of myocardial infarction. The gated SPECT studies were performed using a 2-day stress/gated rest Tc-99m sestamibi protocol. All patient studies were processed using CAFU and quantitative-gated SPECT (QGS), the two software packages for quantification of gated SPECT images. The lower normal limits for EF were higher for CAFU compared with QGS for both women (59% versus 53%) and men (54% versus 47%). The upper normal limits for EDV were also higher for CAFU compared with QGS for both women (133 versus 107 ml) and men (182 versus 161 ml). The differences between the software packages were small for ESV (women 44 versus 44 ml; men 69 versus 74 ml). Gender-specific normal limits need to be applied for LV EF and volumes determined by gated SPECT. Separate criteria for abnormal LV EF and EDV need to be used for women and men depending on the software package used.

## Introduction

We recently presented CAFU, a new method for quantification of left ventricular (LV) volumes and ejection fraction (EF) from myocardial perfusion, gated single photon emission computed tomography (SPECT) images (Lomsky *et al.*, 2005). The innovative approach with CAFU, compared with previously presented methods, is the use of the active shape algorithm. With this technique a non-geometrical, heart-shaped model is fitted to the left ventricle in the three-dimensional (3D) image space. The method has been compared with the most widely used software package for quantification of gated SPECT images, the Cedar-Sinai quantitative-gated SPECT (QGS) program (Germano *et al.*, 1995), in a group of 316 consecutive patients (Lomsky *et al.*, 2006). The results showed that CAFU end-diastolic volume (EDV) measurements were 29 ml higher on average than the corresponding QGS measurements, with the differences being larger with larger volumes. The CAFU EF measurements were four points higher on average than the corresponding QGS measurements, while the CAFU end-systolic volume (ESV) measurements were 6 ml higher on average than the corresponding QGS measurements. Similar studies have been performed, in which two or more software packages have been compared and differences similar to those observed here have been reported (Nakajima *et al.*, 2001; Lum & Coel, 2003; Schaefer *et al.*, 2005). For example, Schaefer *et al.* (2005) compared QGS with 4D-MSPECT and Emory Cardiac Toolbox and found differences in EF measurements of six and nine points, respectively.

In the clinical situation, LV volumes and EF measurements for a specific patient are classified as normal or abnormal based on established normal limits. There are few studies presenting such normal limits and most of them relate to the QGS software package (Ababneh *et al.*, 2000; Rozanski *et al.*, 2000; Sharir *et al.*, 2006). One approach to establishing normal limits for new quantification software is to take the normal limits presented for QGS from one study and adjust them to the differences found between the new and the QGS software package from another study. A problem with this approach would be that the differences reported in a comparison study are based on both normal and abnormal left ventricles and these may not be representative of the differences found in a normal population. As mentioned above, differences between EDV measurements from the QGS and the CAFU software packages have been reported to be larger with larger volumes. The purpose of this study was therefore to establish gender-related normal limits for LV volumes and EF for CAFU, based on a population of normal patients, and to compare them with the corresponding limits for QGS.

## Materials and methods

### Study population

A total of 185 patients, 122 women and 63 men, were retrospectively selected from a consecutive series of patients examined between 15 September 2004 and 14 September 2006 at Sahlgrenska University Hospital in Gothenburg for coronary artery disease (CAD) or for the management of known CAD. Patients were included in the study group if they had normal or probably normal results with stress and rest perfusion imaging and if the combined evaluation of stress/rest non-gated images and gated rest images was normal or probably normal with regard to occurrence of myocardial infarction (MI). One experienced physician interpreted all examination results during the study period. The interpretations were done at the time of clinical reporting, and clinical information and the results of the stress test were available to the physician. The QGS and Cequal software packages were used to aid visual interpretation (Van Train *et al.*, 1993; Germano *et al.*, 1995). Patients with documented hypertension, diabetes, CAD, MI, cardiomyopathy, heart failure, previous revascularization, as well as electrocardiogram (ECG) signs or suspicion of MI, pre-excitation and left bundle branch block (LBBB) were excluded. Patients were neither included nor excluded on the basis of visual or quantitative analysis of global LV function from the gated SPECT images. Regional LV motion and thickening was included in the analysis of perfusion defects compatible with MI. Patients with incomplete data and studies with technical problems were excluded. The mean age was 59·4 ± 9·1 (range 41–83) years for women and 56·6 ± 10·8 (range 31–78) years for men. The women had a mean body surface area (BSA) of 1·74 ± 0·14 (1·27–2·10) m^2^ and the men of 2·02 ± 0·18 (range 1·65–2·50) m^2^. The study was approved by the Research Ethics Committee at Gothenburg University.

### Radionuclide imaging

The gated SPECT studies were performed using a 2-day non-gated stress/gated rest Tc-99m sestamibi protocol. Patients were stressed using either maximal exercise, a symptom-limited ergometry test or pharmacological test with adenosine. The exercise was continued for at least 2 min after the injection of the tracer and the adenosine infusion at least 2·5 min after the injection of the tracer. Stress and rest acquisition began about 60 min after the injection of 600 MBq Tc-99m sestamibi. Images were acquired with two different dual-head SPECT cameras (Millennium VG, GE Healthcare, Milwaukee, WY, USA) equipped with a low-energy high-resolution collimator.

Acquisition, with the patient in the supine position, was done in step-and-shoot mode using circular acquisition and a 64 × 64 matrix, zoom factor 1·28, pixel size 6·9 mm, with 60 projections over 180°, 40 s per projection. In patients weighing >90 kg the acquisition time per projection was increased to 55 s. During the rest acquisition the patient was monitored with a three-lead ECG. The acceptance window was opened to ±20% of the predefined R–R interval. Other beats were rejected. Each R–R interval was divided into eight equal time intervals. Gated SPECT acquisition was performed at the same time as ungated routine SPECT acquisition. An automatic motion-correction program was applied in studies showing patient motion during acquisition. Tomographic reconstruction of non-gated data was performed using filtered back-projection with a Butterworth filter with a critical frequency of 0·52 cycles cm^−1^ and order 5. The reconstruction of gated data was done using filtered back-projection with a Butterworth filter with a critical frequency of 0·40 cycles cm^−1^ and order 10. No attenuation or scatter correction was used.

### Quantitative analysis of gated SPECT images

The short-axis images from all patient studies were processed using the two software packages for quantification of gated SPECT images, CAFU (Lomsky *et al.*, 2005) and QGS (Germano *et al.*, 1995). ESV, EDV and EF were automatically calculated. For the calculations of ESV index (ESVi) and EDV index (EDVi), the ESV and EDV values, respectively, were divided by the BSA.

The CAFU program has recently been presented by our group as a new method for automated quantification of gated SPECT images. The method is based on the active shape algorithm. The search for and delineation of the left ventricle in the SPECT images is based on a heart-shaped LV model. In an iterative process, the model is adjusted to optimize the fit with the image data. The method has been presented elsewhere in detail (Lomsky *et al.*, 2005).

The QGS program was used for comparison with CAFU. The images from both cameras were processed on the same workstation (Entegra workstation, GE Healthcare, Milwaukee, WY, USA) using the QGS program. This program automatically identifies the epi- and endocardial contours for each of the sets of short-axis slices in the cardiac cycle to calculate volume changes.

### Statistical analysis

All statistical analyses were conducted using Microsoft Office Excel 2003. We considered *P* < 0·05 as statistically significant. In the comparisons of mean values, we used paired *t*-test to compare the two different methods and unpaired *t*-test with heterogeneous variances to compare male and female patients. The LV volumes and EF were approximately normally distributed, and consequently, normal limits were defined as the mean values +2 SDs for the volumes and the mean values - 2 SDs for EF. We calculated 95% CIs for the normal limits. We regarded CIs for the two methods that did not overlap as significantly different. Note that this is a conservative approach for paired data.

## Results

Normal gated SPECT values for LV volumes and EF, calculated using CAFU and QGS, are summarized in Table 1. EDV, EDVi and EF were significantly higher for CAFU compared with QGS for both women and men. ESV and ESVi were significantly higher for CAFU compared with QGS for women, but not for men. The variances for LV volumes and EF were generally of a similar magnitude for CAFU and QGS. LV volumes were significantly smaller and EF was significantly higher in women compared with men.

**Table 1:** Mean values of left ventricular (LV) volumes and ejection fraction (EF) for CAFU and quantitative-gated SPECT (QGS).

	CAFU	QGS	
Women (*n* = 122)			
ESV (ml)	28 (8)	25 (10)	*P* < 0·001
ESVi (ml m^−2^)	16 (5)	14 (5)	*P* < 0·001
EDV (ml)	99 (17)	75 (16)	*P* < 0·001
EDVi (ml m^−2^)	57 (9)	43 (9)	*P* < 0·001
EF (%)	72 (6)	68 (7)	*P* < 0·001
Men (*n* = 63)			
ESV (ml)	46 (12)	47 (14)	*P* > 0·30
ESVi (ml m^−2^)	23 (5)	23 (7)	*P* > 0·30
EDV (ml)	132 (25)	112 (24)	*P* < 0·001
EDVi (ml m^−2^)	66 (12)	56 (12)	*P* < 0·001
EF (%)	65 (6)	59 (6)	*P* < 0·001

Values are given in means (±SD).

The normal limits and corresponding 95% CIs are presented in Table 2. The table also includes the normal limits from three previously presented studies (Ababneh, 2000; Rozanski, 2000; Sharir, 2006). For EDV and EF, there was no overlap between the 95% CI for CAFU and QGS for women and men. These findings indicate that different normal limits should be used for women and men as well as for different quantification software packages. For ESV, there was no overlap between the 95% CI between women and men, indicating that separate normal limits should be used. There was no difference between CAFU and QGS in normal ESV limits for women and only a small difference for men (69 versus 74 ml), with overlapping CIs indicating that similar normal limits could be used.

**Table 2:** Normal limits of gated SPECT variables for CAFU and quantitative-gated SPECT (QGS), based on this investigation and three previously presented studies.

	CAFU	QGS	QGS, Ababneh *et al.* (2000)	QGS, Sharir *et al.* (2006)	Simpson's rule technique, Rozanski *et al.* (2000)
Women	(*n* = 122)	(*n* = 122)	(*n* = 124)	(*n* = 597)	(*n* = 100)
ESV (ml)	44 (41–46)	44 (41–47)	40	46	47
ESVi (ml m^−2^)	26 (24–27)	25 (23–27)	20	27	26
EDV (ml)	133 (128–138)	107 (102–111)	91	102	106
EDVi (ml m^−2^)	74 (72–77)	60 (58–63)	48	60	57
EF (%)	59 (57–61)	53 (51–55)	51	51	49
Men	(*n* = 63)	(*n* = 63)	(*n* = 116)	(*n* = 824)	(*n* = 78)
ESV (ml)	69 (64–74)	74 (68–80)	55	75	78
ESVi (ml m^−2^)	33 (31–36)	36 (34–39)	27	39	38
EDV (ml)	182 (172–193)	161 (150–171)	119	149	157
EDVi (ml m^−2^)	90 (84–95)	79 (74–84)	56	75	76
EF (%)	54 (52–56)	47 (45–50)	48	43	41

EDV, end-diastolic volume; EDVi, end-diastolic volume index; EF, ejection fraction; ESV, end-systolic volume; ESVi, end-systolic volume index. Upper limit of normal, calculated as the mean +2 SDs for ESV, ESVi, EDV and EDVi. Lower limit of normal, calculated as the mean -2 SDs for EF. 95% CIs of the limits are presented for this study population.

## Discussion

Gender-related normal limits for LV volumes and EF from gated SPECT were established for the CAFU method. The differences between these normal limits and the corresponding limits for QGS are slightly higher for EF (women 6%; men 7%) compared with the previously presented EF difference between the two methods in a mixed patient population (4%) (Lomsky *et al.*, 2006). The relation was the opposite for EDV, where the difference between the methods in the mixed population was 29 ml and the differences between the normal limits were 26 ml for women and 21 ml for men. Ficaro *et al.* (2003) hypothized that differences between QGS and another software package for determining LV EF could be explained by the QGS algorithms constraining the LV basal motion. The CAFU algorithm does not constrain the LV basal motion, which could be one explanation for the higher EDV values for CAFU compared with QGS in this study.

For men, the normal ESV limit was 5 ml higher for QGS compared with CAFU, while in the mixed population of patients CAFU showed 4 ml higher ESV values on average. These findings can be explained by larger differences in measurements between the two software packages for larger volumes. The mixed group of patients (Lomsky *et al.*, 2006) included a consecutive group of patients referred to myocardial perfusion scintigraphy during a 6 months period. Patients with large and severe perfusion defects as well as those with normal perfusion were included in that study.

The QGS normal limits for EF in this material (women 53%; men 47%) were close to those presented by Ababneh *et al.* (2000) (women 51%; men 48%), while Sharir *et al.* (2006) reported lower EF limits for men (43%) (Table 2). Rozanski *et al.* (2000), who used a modified Simpson's rule technique, also found a lower normal limit for men (41%). The QGS normal limits for EDV and ESV in this study, on the other hand, were close to those presented by Sharir *et al.* and Rozanski *et al.*, while Ababneh and colleagues reported considerably lower EDV and ESV limits for both women and men. The differences between our QGS limits and those of Ababneh *et al.* and Sharir *et al.*, on the one hand, and those presented by Rozanski *et al.*, on the other, could at least in part be explained by the different software used. Moreover, Ababneh *et al.* included patients with diabetes mellitus, hypertension, prior revascularization or a combination of these factors, patients who were excluded from the present study. These factors could, however, hardly explain why Ababneh *et al.* found lower EDV and ESV values for both women and men.

A large group of randomly selected healthy volunteers would probably be a better study group for establishing normal limits. This type of reference population is rarely available in nuclear medicine and ‘healthy’ patients are often used instead. This approach can be criticized, since subjects referred to myocardial perfusion imaging may have some reasons for the referral, which may not be found at the examination, e.g. microvascular disease or non-cardiac disease, indicating that they may not be representative of a healthy reference population. In this study we excluded patients with documented hypertension, diabetes, CAD, MI, heart failure, previous revascularization, cardiomyopathy, ECG signs or suspicion of MI, LBBB, and pre-excitation abnormal ECG at rest. The patients with diabetes, hypertension and prior revascularization are likely to represent a ‘less well’ part of the reference population, leading to excessively broad normal limits. On the other hand, applying very rigorous exclusion criteria may lead to a reference population that represents a ‘too healthy’ part of the population. We believe that the inclusion and exclusion criteria in this study represent a reasonable balance in order to have a relevant reference population for establishing normal limits.

The number of women was nearly twice as many as the number of men in this study. This is a somewhat surprising finding, since the study group was selected from a consecutive series of patients examined for CAD or for the management of known CAD and the numbers of women and men were about the same in the total group. There could be a selection bias in that more women with low likelihood of CAD, but with unspecific ECG changes at a preceding exercise test, were referred to myocardial perfusion scintigraphy in order to rule out significant CAD.

In conclusion, gender-specific normal limits need to be applied for LV EF and volumes determined by gated SPECT. Separate criteria for abnormal LV EF and EDV need to be used for both women and men, depending on the software package used.
